# *Laminaria japonica* Suppresses the Atopic Dermatitis-Like Responses in NC/Nga Mice and Inflamed HaCaT Keratinocytes via the Downregulation of STAT1

**DOI:** 10.3390/nu12113238

**Published:** 2020-10-23

**Authors:** Youn-Hwan Hwang, Hyun-Kyung Song, Ami Lee, Hyunil Ha, Taesoo Kim

**Affiliations:** 1 Herbal Medicine Research Division, Korea Institute of Oriental Medicine, 1672 Yuseong-daero, Yuseong-gu, Daejeon 34054, Korea; hyhhwang@kiom.re.kr (Y.-H.H.); gusrud1654@kiom.re.kr (H.-K.S.); dmb01367@kiom.re.kr (A.L.); 2 Korean Convergence Medicine Major KIOM, University of Science & Technology (UST), 1672 Yuseongdae-ro, Yuseong-gu, Daejeon 34054, Korea

**Keywords:** *Laminaria japonica*, atopic dermatitis, HaCaT cells, NC/Nga mice

## Abstract

Atopic dermatitis (AD) is a skin allergy accompanied by acute and chronic dermal inflammation. In traditional oriental medicine, *Laminaria japonica* has been used to treat various diseases, including inflammatory diseases. Therefore, to determine the therapeutic potential of *L. japonica* against AD, we investigated the inhibitory effects of *L. japonica* water extract (LJWE) on the inflammatory mediators and AD-like skin lesions. We determined the cell viability of LJWE-treated HaCaT cells using the cell counting kit-8 assay and the levels of inflammatory cytokines using cytometric bead array kits. Additionally, we analyzed the modulatory effects of LJWE on the signaling pathways in tumor necrosis factor-α/interferon-γ-stimulated HaCaT cells via Western blotting. Furthermore, we determined the in vivo effect of LJWE on NC/Nga mice and found that LJWE remarkably improved the skin moisture, reduced dermatitis severity, and inhibited the overproduction of inflammatory mediators in 2,4-dinitrochlorobenzene-sensitized NC/Nga mice. We also observed that LJWE inhibits the expression of inflammatory chemokines in human keratinocytes by downregulating the p38 mitogen-activated protein kinase signaling pathway and activating the signal transducer and activator of transcription 1. In conclusion, LJWE has the therapeutic potential against AD by healing AD-like skin lesions, and suppressing inflammatory mediators and major signaling molecules.

## 1. Introduction

Atopic dermatitis (AD), a skin allergy that affects infants and adults, is characterized by clinical symptoms such as redness, dryness, itchiness, and the thickening of the inflamed skin lesions [1,2]. The pathogenesis of AD is affected by a variety of factors, including genetics, environment, psychological state, host immune dysfunction, and defective epidermal barriers [1]. Although the pathogenesis of AD is not fully clarified, many researchers have found that AD is associated with a defective skin barrier and leads to allergic responses in inflamed keratinocytes [3]. In the skin lesion of acute AD, the secretion of IL-4, IL-5, and IL-13 by Th-2 cells, and IgE production by B cells, are increased [4]. However, the increased productions of interferon (IFN)-γ and tumor necrosis factor (TNF)-α are observed in the chronic phase [5]. In particular, keratinocytes stimulated by pro-inflammatory cytokines secrete inflammatory mediators under the regulation of nuclear factor-kappa B (NF-κB), mitogen-activated protein kinases (MAPKs), and signal transducer and activator of transcription (STAT), leading to the infiltration of monocytes, mast cells, and T lymphocytes and the stimulation of inflammatory response in the dermis [6,7,8]. Although current therapies recommend the use of anti-inflammatory agents (e.g., corticosteroids), immunosuppressants (e.g., cyclosporin, methotrexate, and azathioprine), and biologics which are effective [9], they can exert adverse side effects and prove to be inefficient in patients who are drug tolerant. Therefore, the development and application of herbal medicines and their derivatives, as complementary and alternative candidates, to relieve the symptoms of AD are increasing because of their long-term use and effectiveness against AD [10].

*Laminaria japonica*, a member of brown algae, is known as “Gonpo” or “Dasima” in Korea, “Kunbu” or “Haidai” in Chinese, and “Kelp” in Europe and North America [9]. In traditional oriental medicines, *L. japonica* has been used to treat scrofula, goiter, edema, testicular pain and swelling, chronic bronchitis, and pulmonary tuberculosis [11,12]. Recently, it has been demonstrated that *L. japonica* has various biological activities such as anti-tumor, anti-coagulation, anti-viral; it also induces hypoglycemia and hypolipidemia. The in vitro and in vivo immunomodulatory effects of *L. japonica* and its components are well known [11,13,14,15]. In particular, a previous study showed that *L. japonica* inhibited the overproduction of ultraviolet B -induced inflammatory cytokines and mediators in human keratinocytes [16]. Although *L. japonica* is a potent candidate against AD, its anti-AD properties remain undetermined. In this study, we investigated the inhibitory effects of *L. japonica* water extract (LJWE) on the overproduction of inflammatory cytokines in TNF-α and IFN-γ-stimulated human keratinocytes, and assessed the beneficial effects of LJWE in AD-like skin lesions in NC/Nga mice.

## 2. Materials and Methods

### 2.1. Materials

Dulbecco’s modified Eagle’s medium (DMEM) was obtained from Hyclone (Logan, UT, USA). Phosphate-buffered saline, fetal bovine serum (FBS), penicillin, and streptomycin were obtained from Gibco-BRL (Gaithersburg, MD, USA). The Cell Counting Kit-8 (CCK-8) was purchased from Dojindo Molecular Technologies Inc. (Rockville, MD, USA). The bicinchoninic acid assay (BCA) kit, IFN-γ and TNF-α were purchased from Thermo Fisher Scientific (Waltham, MA, USA). L-leucine, isoleucine, phenylalanine, tryptophan, linolenic acid, palmitic acid and stearic acid were purchased from Cayman chemical (Ann Arbor, MI, USA). The 15-hydroxy-5,8,11,13-eicosatetraenoic acid, stearidonic acid, eicosapentaenoic acid, arachidonic acid, and linoleic acid were obtained from Targetmol (Boston, MA, USA). MS-grade acetonitrile, water and formic acid were purchased from Thermo Fisher Scientific (Waltham, MA, USA). The p-STAT1, STAT1, p-IκBα, IκBα, p-p38, p38, p-ERK, ERK, p-JNK, JNK, p65, and β-actin antibodies were purchased from Cell Signaling Technology (Beverly, MA, USA). p50 and anti-proliferating cell nuclear antigen antibodies were obtained from Santa Cruz Biotechnology (Santa Cruz, CA, USA).

### 2.2. Preparation and Chemical Profiling of LJWE

LJWE was obtained from the KOCBiotech (Daejeon, Republic of Korea) and stored in the herbarium (voucher number #KW-6) of the Herbal Medicine Research Division. Dried *L. japonica* (1 kg) was extracted with distilled water (10 L, *v*/*v*) under reflux for 3 h at 100 °C and lyophilized after filtration. Brownish LJWE powder was stored at −20 °C until further use. To determine the constituents of LJWE, we performed an ultra-performance liquid chromatography-tandem mass spectrometry (UPLC–MS/MS) analysis, according to previously reported methods [17,18]. Briefly, a Dionex UltiMate 3000 system coupled with a Thermo Q-Exactive mass spectrometer was used. Chromatographic separation was achieved by an Acquity BEH C18 column (100 × 2.1 mm, 1.7 μm) with acetonitrile, 0.1% formic acid in water. Data acquisition and analysis were performed using Xcalibur, TraceFinder 3.2, and Compound Discoverer 3.1 softwares (Thermo Fisher Scientific, Waltham, MA, USA).

### 2.3. Cell Viability and Treatments

HaCaT cells, from an immortalized human skin keratinocyte cell line, were purchased from Elabscience (Houston, TX, USA). The cells were cultured in DMEM supplemented with 10% FBS, 100 μg/mL streptomycin, and 100 U/mL penicillin at 37 °C in a humidified incubator with 5% CO_2_. To determine the cell viability, the cells were seeded at a density of 1 × 10^4^ cells/well in a 96-well plate and incubated with LJWE (25–200 μg/mL) for 24 h. The viability of the LJWE-treated cells was determined using the CCK-8 assay, according to the manufacturer’s guidelines. For measuring the chemokine levels, the cells (1 × 10^4^ cells/well in a 96-well plate) were pretreated with LJWE for 1 h and incubated with or without IFN-γ and TNF-α (each 10 ng/mL) for 24 h. After incubation, the levels of interleukin (IL)-8, regulated on the activation of normal T-cell expressed and secreted (RANTES), and thymus and activation-regulated chemokine (TARC) in culture medium were determined.

### 2.4. Western Blotting

HaCaT cells were seeded in 10 cm dishes and cultured until approximately 70% confluency was obtained. The cells were pretreated with LJWE for 1 h and incubated with or without TNF-α/IFN-γ (each 10 ng/mL) for 3 h. Whole cell lysates and nuclear proteins were extracted using cold radioimmunoprecipitation assay buffer with protease and phosphatase inhibitors (Biosesang, Seongnam, Korea) and NE-PER* nuclear and cytoplasmic extraction reagents (Pierce Biotechnology, Rockford, IL, USA), respectively. The protein content of the cell was quantitated using the BCA kit. Equal amounts of the extracted protein (20 μg) were separated using 4–15% Mini-PROTEAN* TGX™ Precast Protein Gels (Bio-Rad, Hercules, CA, USA) via electrophoresis and then transferred on to Fluoro Trans* polyvinylidene fluoride membrane (Pall Corporation, Dreieich, Germany). The membranes were blocked with 5% skim milk (Sigma, St. Louis, MO, USA) or 3% bovine serum albumin (MP Biomedicals, Irvine, CA, USA) for 2 h, and the primary antibodies (1:1000 dilution) were incubated with the membrane in blocking solution at 4 °C overnight, followed by the incubation with secondary antibodies (horseradish peroxidase-conjugated anti-IgG) at 4 °C for 1 h. ChemiDoc Imaging System (Bio-Rad, Hercules, CA, USA) was used to detect protein expression.

### 2.5. Animal Study

Female NC/Nga mice (5 week-old) were purchased from Japan SLC (Shizuoka, Japan). The mice were acclimatized under standard housing conditions (22 ± 2 °C and 50 ± 10% humidity with a 12/12 h light/dark cycle) for 7 days. Water and food were provided ad libitum to the mice. All procedures involving animals were approved by the Institutional Animal Care and Use Committee of the KPC lab (Gwangju, Republic of Korea; approved number, KPC-P160006). The mice were exposed to 2,4-dinitrochlorobenzene (DNCB; Sigma-Aldrich, St. Louis, MO, USA) to induce AD. Briefly, to induce AD-like skin lesions, 0.2 mL of 1% DNCB dissolved in acetone-olive oil (3:1, *v*/*v*) solution after the the complete removal of dorsal coat hair was used. Subsequently, DNCB-sensitized mice were challenged by applying 0.8% DNCB thrice per week, for 2 weeks, on the same region of dorsal skin. The mice were randomly divided into five groups (*n* = 7), normal control (vehicle alone), negative control (DNCB alone), positive control (DNCB + 5 mg/kg of prednisolone), low-dose LJWE (LJWE L; DNCB + 100 mg/kg of LJWE), and high-dose LJWE (LJWE H; DNCB + 100 mg/kg of LJWE) groups. LJWE and prednisolone was dissolved in distilled water (vehicle) and the dose volume administered was 10 mL/kg. At the end of the experimental period, the mice were sacrificed using CO_2_ anesthesia and the serum samples were collected for further analyses.

### 2.6. Analysis of Dermatitis Severity, Scratching Behavior, and Trans-Epidermal Water Loss (TEWL)

The severity of skin lesions was blindly graded every two weeks, for 4 weeks, according to the method reported by Kang et al. [19]. The total severity index of dermatitis was defined as the sum of individual scores graded as follows: 0 (none), 1 (mild), 2 (moderate), and 3 (severe) for each of the five symptoms: erythema, itchiness/dryness, edema/hematoma, excoriation/erosion, and lichenification. The scratching frequency was recorded using a digital camera, and the number of scratching behaviors with the hind limbs on the dorsal skin was counted for 20 min. Trans-epidermal water loss (TEWL) from mouse dorsal skin was measured every two weeks, for 4 weeks, using Tewameter* TM300 (CK electronic GmbH, Germany) and data were recorded under specific conditions of 24–25 °C and 50–60% humidity after achieving stabilization at approximately 30–45 s.

### 2.7. Estimation of Inflammatory Chemokines

The chemokines (TARC, RANTES, and IL-8) present in the culture medium of HaCaT cells and the chemokines (RANTES and TARC) present in the mice were measured using cytometric bead array kits (LEGENDplex, Biolegend, San Diego, CA, USA) according to the manufacturer’s instructions. The levels of IgE were estimated using a commercial ELISA kit (mouse IgE ELISA MAX™, Biolegend, San Diego, CA, USA).

### 2.8. Statistical Analysis

Data are represented as the mean ± standard error of the mean (SEM). Most data using one-way analysis of variance (ANOVA) with Dunnett’s post-hoc test, or the Mann–Whitney U test with the Kruskal–Wallis test were analyzed via Prism (GraphPad, San Diego, CA, USA). Western blot analysis was compared using the unpaired two-tailed *t* test.

## 3. Results and Discussion

### 3.1. Inhibitory Effects of LJWE on the Overproduction of Inflammatory Chemokines in TNF-α/IFN-γ-Stimulated HaCaT Cells

Keratinocytes largely form epidermis and maintain skin homeostasis via the secretion of inflammatory chemokines and cytokines, thereby leading to the recruitment of immune cells. TNF-α and IFN-γ can synergistically induce chemokine production in keratinocytes [20]. In the pathogenesis of AD, the stimulation of keratinocytes with TNF-α/IFN-γ results in the production of inflammatory chemokines, including IL-8, RANTES, and TARC [5,21,22,23]. These chemokines aggravate dermal inflammation by giving rise to AD-like skin lesions that are associated with the development and severity of AD. Stimulated keratinocytes secrete IL-8 resulting in the infiltration of T cells and neutrophils into the epidermis [24,25]. TARC and RANTES secreted by keratinocytes play an important role in the activation of macrophages and infiltration of T helper 2 cells in the inflamed tissues [5]. Therefore, we investigated whether LJWE could suppress these chemokines in HaCaT human keratinocyte cells co-stimulated with TNF-α/IFN-γ.

**Figure 1:**
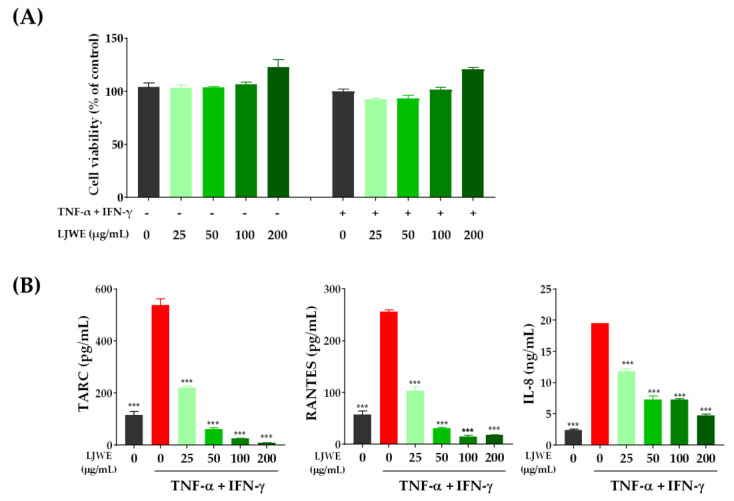
*Laminaria japonica* water extract (LJWE) inhibits the expression of inflammatory chemokines in tumor necrosis factor (TNF)-α/interferon (IFN)-γ-stimulated HaCaT cells. (**A**) Cell viability in the presence of LJWE. The cells were treated with 0–200 μg/mL of LJWE with (+) or without (−) TNF-α/IFN-γ-stimulation for 24 h, and the cell viability was measured using the Cell Counting Kit-8 assay. (**B**) The inhibitory effects of LJWE on the overproduction of interleukin (IL)-8, regulated on activation normal T-cell expressed and secreted (RANTES), and thymus and activation-regulated chemokine (TARC). *** *p* < 0.001 vs. control treated with vehicle.

To determine the optimal effective range of LJWE, we treated HaCaT cells with varying concentrations of LJWE ranging from 25 to 200 μg/mL for 24 h. However, no remarkable changes in the cell viability were observed up to the concentration of 200 μg/mL regardless of the co-stimulation of TNF-α/IFN-γ (Figure 1A). We then examined the inhibitory effect of LJWE on the expression of inflammatory chemokines in TNF-α/IFN-γ-stimulated HaCaT cells. Consistent with the results of previous reports [24,26], co-stimulation with TNF-α/IFN-γ in HaCaT cells significantly increased the expression of TARC, RANTES, and IL-8 compared to the vehicle control (*p* < 0.001). Additionally, LJWE significantly inhibited the expression of TARC, RANTES, and IL-8 in a dose-dependent manner (*p* < 0.001) (Figure 1B). These data suggest that LJWE can suppress the expression of inflammatory chemokines by TNF-α/IFN-γ co-stimulation in human keratinocytes.

### 3.2. Effects of LJWE on the Expression of MAPK/NF-κB/STAT1 in TNF-α/IFN-γ-Stimulated HaCaT Cells

**Figure 2:**
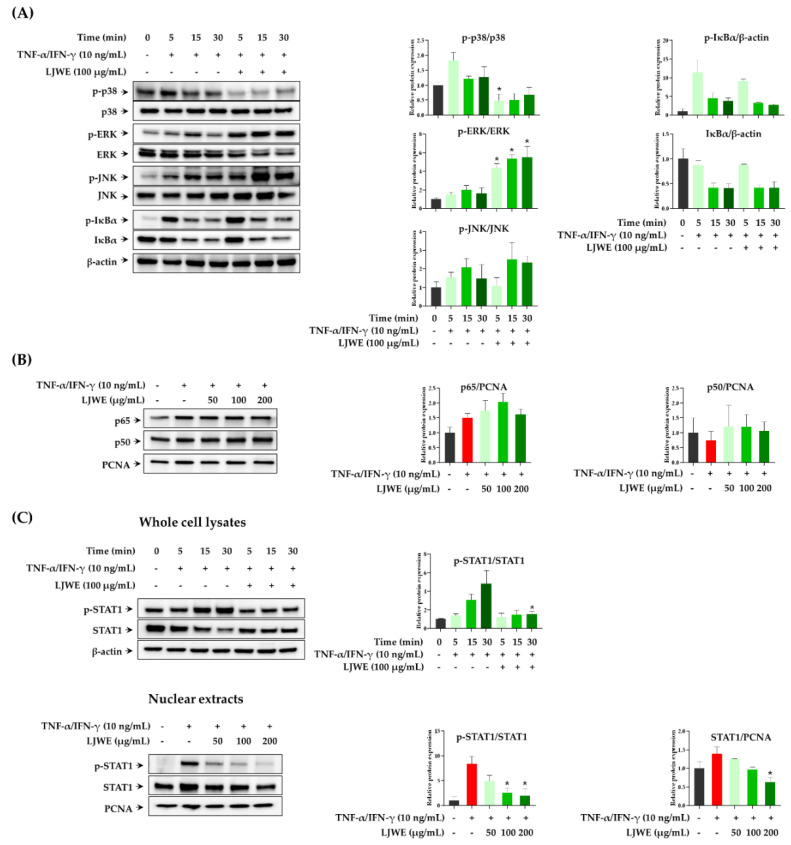
Modulatory effects of LJWE on mitogen-activated protein kinases (MAPKs), nuclear factor-kappa B (NF-κB), and signal transducer and activator of transcription 1 (STAT1) signaling pathways in tumor necrosis factor (TNF)-α/interferon (IFN)-γ-stimulated HaCaT cells. The cells were pretreated with (+) or without (−) LJWE for 1 h and incubated in the presence (+) or absence (−) of TNF-α/IFN-γ (each 10 ng/mL) for 5, 15, and 30 min. Western blotting was performed to determine the phosphorylation and degradation of p38, extracellular signal-regulated kinase (ERK) and c-Jun N-terminal kinase (JNK), Inhibitor kappa B-alpha (IκBα), and STAT1 in whole cell lysates. The translocation of NF-κB and STAT1 was determined 3 h post-TNF-α/IFN-γ treatment. (**A**) Effects of LJWE on the phosphorylation and degradation of MAPKs in whole cell lysates. (**B**) Effects of LJWE on the nuclear translocation of NF-κB. (**C**) Effects of LJWE on the activation (upper panel) and nuclear translocation (lower panel) of STAT1. * *p* < 0.05 vs. control treated with vehicle. PCNA, proliferating cell nuclear antigen.

The expression of inflammatory chemokines, including TARC, RANTES, and IL-8 in TNF-α/IFN-γ-stimulated HaCaT cells was primarily regulated by MAPKs, NF-κB, and/or STAT signaling pathways [24,27,28]. NF-κB and STAT1, the important transcription factors that are activated by pro-inflammatory cytokines, that are present in the cytoplasm translocate into the nucleus and lead to the expression of several inflammatory chemokine and cytokine genes [29,30]. TNF-α/IFN-γ stimulation in HaCaT cells activates intracellular MAPK signaling pathways, thereby inducing the activation and translocation of NF-κB and STAT1. Thus, we first evaluated the modulatory effect of LJWE on the TNF-α/IFN-γ-induced phosphorylation of MAPKs (p38, ERK, JNK, and IκBα) in the signaling pathway of HaCaT cells. We observed that LJWE inhibited the phosphorylation of p38-MAPK in TNF-α/IFN-γ-stimulated HaCaT cells, while LJWE did not inhibit the phosphorylation or degradation of ERK, JNK, and IκBα (Figure 2A). Similar to our results, several studies found that p38-MAPK, but not ERK and JNK, were involved in the TNF-α/IFN-γ-induced expression of inflammatory chemokines in HaCaT cells [27,31,32]. We then determined whether LJWE could inhibit the activation and intranuclear translocation of NF-κB and STAT1 after TNF-α/IFN-γ-stimulation. In accordance with the previous reports, co-stimulation with TNF-α/IFN-γ increased the intranuclear translocation of NF-κB p65, whilst LJWE did not affect this phenomenon (Figure 2B). We determined that the phosphorylation of STAT1 was inhibited by LJWE in LJWE-treated HaCaT cells (Figure 2C, upper panel). Furthermore, LJWE inhibited the intranuclear translocation of STAT1 in a dose-dependent manner (Figure 2C, lower panel). Kwon et al. [27] demonstrated that blocking STAT1 activation through the p38 MAPK pathway inhibited chemokine productions in HaCaT cells using p38-MAPK inhibitor. These findings indicate that the inhibitory effects of LJWE on the production of inflammatory chemokines in human keratinocytes could be partially contributed by the downregulation of the p38-MAPK signaling pathway, and STAT1 activation.

### 3.3. Healing of DNCB-Induced AD-Like Skin Lesions by LJWE in NC/Nga Mice

**Figure 3:**
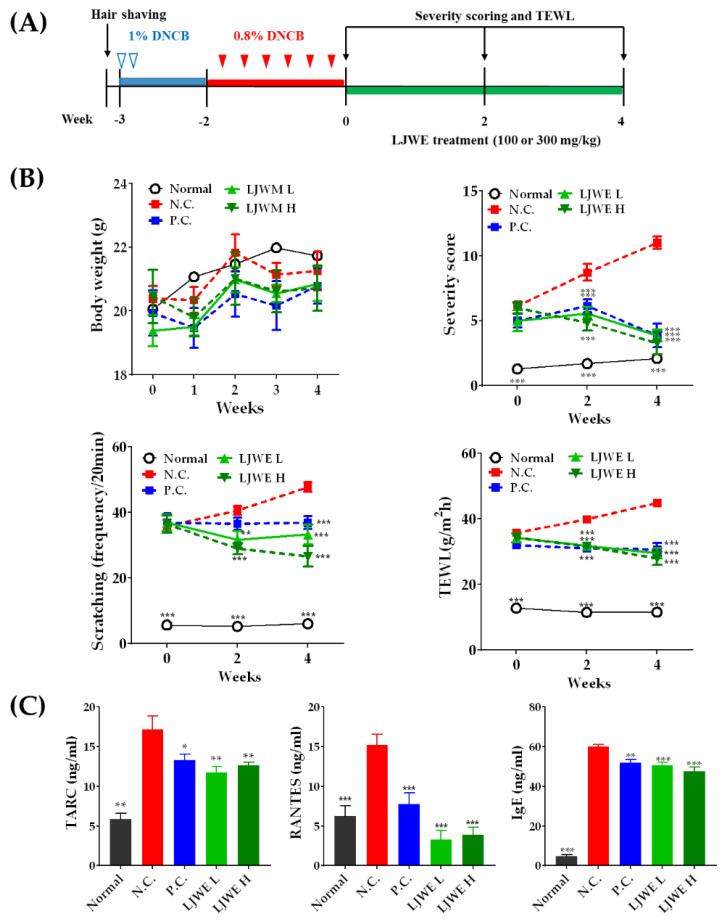
LJWE ameliorates atopic dermatitis (AD)-like skin lesions in NC/Nga mice. (**A**) Schematic representation of the experiment investigating the effect of LJWE on AD-like skin lesions induced by 2,4-dinitrochlorobenzene (DNCB). (**B**) Changes in the body weight, severity score of dermatitis, and trans-epidermal water loss (TEWL) during the experimental periods. (**C**) Inhibitory effects of LJWE on the overproduction of TARC, RANTES and IgE. Normal (no DNCB/vehicle); negative control (N.C., DNCB/vehicle); positive control (P.C., DNCB/5 mg/kg of prednisolone); low-dose LJWE (LJWE L; DNCB + 100 mg/kg per day of LJWE); high-dose LJWE (LJWE H; DNCB + 300 mg/kg per day of LJWE). All data are expressed as the mean ± standard error of the mean (SEM) (*n* = 7). * *p* < 0.05, ** *p* < 0.01, *** *p* < 0.001 versus N.C.

NC/Nga mice have been widely used as an experimental model for the investigation of the pathogenesis of AD and the development of anti-AD drug candidates. The pathological changes in the skin of NC/Nga mice mimic AD by rapidly developing symptoms such as itchiness, erythema, erosive lesions with edema, and hemorrhage on the skin [33,34]. The NC/Nga mice gradually increased their IgE production, whereas TARC and its receptor were highly expressed in the skin lesions of the mice. Moreover, DNCB, a cytotoxic benzene derivative, resulted in AD-like skin lesions in NC/Nga mice. To determine the beneficial effects of LJWE on AD-like skin lesions, DNCB-sensitized NC/Nga mice were administered with prednisolone (5 mg/kg) and LJWE (100 or 300 mg/kg per day) for 4 weeks (Figure 3A). During the experimental period, the body weight of LJWE-treated mice increased as expected (Figure 3B, upper left panel). Repeated exposure to DNCB apparently induced AD-like symptoms, such as erythema, dryness, thickening, and lichenification, whereas the severity of AD was significantly reduced in the LJWE-treated mice in comparison with those in the negative control group (*p* < 0.001, Figure 3B, upper right panel). We monitored the frequency of dorsal skin scratching to evaluate the antipruritic effect of LJWE on DNCB-sensitized NC/Nga mice. The frequency of scratching was significantly reduced in the LJWE-treated mice as compared to in the vehicle control (*p* < 0.001, Figure 3B, lower left panel), thereby reducing the severity of AD. We estimated TEWL in DNCB-sensitized NC/Nga mice to examine the effect of LJWE on the functions of skin barriers. Although the DNCB-sensitized group showed a significant increase in TEWL score, the LJWE treatment significantly reduced TEWL at all doses (*p* < 0.001, Figure 3B, lower right panel). Therefore, we found that LJWE improved the health of the skin and its healing in vivo, indicating that LJWE is a potential therapeutic candidate to treat AD.

We measured the levels of inflammatory chemokines (TARC and RANTES) and serum IgE to determine the anti-pruritic and anti-inflammatory effects of LJWE. The TARC, RANTES, and IgE levels in the DNCB-sensitized mice were significantly higher than those in the vehicle group (*p* < 0.001, Figure 3C). The increased expression of TARC and RANTES induced by DNCB decreased post LJWE treatment. Additionally, the LJWE treatment significantly decreased the IgE levels, an important inflammatory mediator, and induced pruritus ((*p* < 0.01, Figure 3C). Several reports have reported elevated serum levels of TARC in patients suffering from AD and that the severity of the disease is closely correlated with chemokine levels [27]. Therefore, TARC was considered to play an important role in the pathogenesis of AD. Furthermore, the elevated serum levels of RANTES that enable eosinophil recruitment were found in the patients suffering from AD [35]. Collectively, these data suggest that the beneficial effects of LJWE may affect the progression of AD-like skin lesions by inhibiting dermal inflammation and itching through the regulation of inflammatory chemokines and the serum IgE levels. However, further studies are required to clarify the precise mechanisms driving the LJWE-mediated healing of skin lesions and modulation of inflammatory cytokines (e.g., IL-1β, IL-6, and IL-31).

### 3.4. Chemical Profiling of LJWE Using UPLC–MS/MS

**Figure 4:**
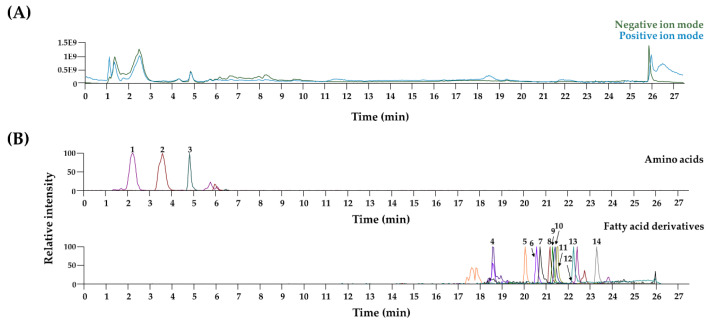
UPLC–tandem MS analysis of LJWE. (**A**) Total ion chromatogram of LJWE. (**B**) Extracted ion chromatograms of LJWE. 1, leucine/isoleucine; 2, phenylalanine; 3, tryptophan; 4, 15-hydroxy-5,8,11,13-eicosatetraenoic acid; 5, stearidonic acid; 6, eicosapentaenoic acid; 7, linolenic acid; 8, hexadecanamide; 9, arachidonic acid; 10, oleamide; 11, linoleic acid; 12, stearamide; 13, palmitic acid; 14, stearic acid.

Most of the identified compounds in LJWE were compared with the retention time and the mass spectrum of reference standards. In cases of hexadecanamide, oleamide, and stearamide, we matched the *m*/*z* and the MS fragment information with mass spectrum databases including mzCloud (www.mzcloud.org) and MassBank of North America (http://mona.fiehnlab.ucdavis.edu). In Figure 4 and Table 1, three amino acids and 11 fatty acid derivatives in LJWE were identified in agreement with previous reports [36]. Several reports demonstrated anti-AD properties of long-chain polyunsaturated fatty acids as indispensable components of a diet [37,38]. Linoleic acid and linolenic acid improve AD-like skin lesion in 2, 4-dinitrofluorobenzene-sensitized mice [39]. Eicosapentaenoic acid can ameliorate allergic dermal inflammation through the suppression of leukotriene B4 [40]. Anti-AD effects of the other compounds have not been found. Collectively, nutraceutical interactions of long-chain polyunsaturated fatty acids in LJWE could attribute to improve AD-like lesions and promote skin healing.

## 4. Conclusions

To the best of our knowledge, this is the first study to investigate the beneficial effects of LJWE, as shown in in vitro and in vivo conditions that mimic AD. LJWE inhibits inflammatory chemokines, improves clinical symptoms, and the severity of the disease and these effects could be attributed to the inhibition of the STAT1 signaling pathway. Taken together, LJWE is an effective nutraceutical that can potentially be used to manage AD. Further studies are needed to ensure the safety and efficiency of LJWE against AD.

## Figures and Tables

**Table 1:** Chemical characterization of LJWE constituents using ultra-performance liquid chromatography-tandem mass spectrometry.

No	R_t_ (min)	Calculated (*m*/*z*)	Estimated (*m*/*z*)	Adduct	Error (ppm)	Formula	MS/MS Fragments (m/z)	Identifications
**Amino acids**								
1	2.2	132.102	132.102	[M + H]^+^	2.683	C_6_H_13_NO_2_	86	L-Leucine/Isoleucine *
2	3.6	166.086	166.087	[M + H]^+^	2.284	C_9_H_11_NO_2_	166, 120	Phenylalanine *
3	4.8	205.097	205.098	[M + H]^+^	1.948	C_11_H_12_N_2_O_2_	188, 159, 146	L-Tryptophan *
**Fatty acid derivatives**								
4	18.6	319.228	319.228	[M − H]^−^	1.736	C_20_H_32_O_3_	319, 301, 257, 219, 175, 113	15-Hydroxy-5,8,11,13 -eicosatetraenoic acid *
5	20.0	275.202	275.202	[M − H]^−^	2.033	C_18_H_28_O_2_	275, 231	Stearidonic acid *
6	20.6	301.217	301.218	[M − H]^−^	1.673	C_20_H_30_O_2_	301, 257, 203	Eicosapentaenoic acid *
7	20.7	277.217	277.218	[M − H]^−^	1.928	C_18_H_30_O_2_	277	Linolenic acid *
8	21.2	256.263	256.264	[M + H]^+^	1.305	C_16_H_33_NO	256, 116, 102, 88	Hexadecanamide
9	21.3	303.233	303.234	[M − H]^−^	2.083	C_20_H_32_O_2_	303, 259	Arachidonic acid *
10	21.4	282.279	282.280	[M + H]^+^	2.069	C_18_H_35_NO	282, 265, 247, 177, 149, 135	Oleamide
11	21.5	279.233	279.234	[M − H]^−^	1.934	C_18_H_32_O_2_	279	Linoleic acid *
12	21.9	284.294	284.295	[M + H]^+^	1.430	C_18_H_37_NO	284, 88	Stearamide
13	22.2	255.233	255.233	[M − H]^−^	1.458	C_16_H_32_O_2_	255	Palmitic acid *
14	23.3	283.264	283.265	[M + H]^+^	1.838	C_18_H_36_O_2_	283	Stearic acid *

Rt, retention time. * Compared with retention time (Rt) and the mass spectrum of reference standards.

## References

[1] Boguniewicz M., Leung D.Y.M. (2011). Atopic dermatitis: A disease of altered skin barrier and immune dysregulation. Immunol. Rev..

[2] Ahn C., Huang W. (2017). Clinical presentation of atopic dermatitis. Adv. Exp. Med. Biol..

[3] Agrawal R., Woodfolk J.A. (2014). Skin barrier defects in atopic dermatitis. Curr. Allergy Asthma Rep..

[4] Choi J.H., Jin S.W., Park B.H., Kim H.G., Khanal T., Han H.J., Hwang Y.P., Choi J.M., Chung Y.C., Hwang S.K. (2013). Cultivated ginseng inhibits 2,4-dinitrochlorobenzene-induced atopic dermatitis-like skin lesions in NC/Nga mice and TNF-α/IFN-γ-induced TARC activation in HaCaT cells. Food Chem. Toxicol..

[5] Nedoszytko B., Sokołowska-Wojdyło M., Ruckemann-Dziurdzińska K., Roszkiewicz J., Nowicki R.J. (2014). Chemokines and cytokines network in the pathogenesis of the inflammatory skin diseases: Atopic dermatitis, psoriasis and skin mastocytosis. Postepy Dermatol. Alergol..

[6] Chen G., Goeddel D.V. (2002). TNF-R1 signaling: A beautiful pathway. Science.

[7] Kim M.Y., Lim Y.Y., Kim H.M., Park Y.M., Kang H., Kim B.J. (2015). Synergistic inhibition of tumor necrosis factor-alpha-stimulated pro-inflammatory cytokine expression in HaCaT cells by a combination of rapamycin and mycophenolic acid. Ann. Dermatol..

[8] Kisseleva T., Bhattacharya S., Braunstein J., Schindler C.W. (2002). Signaling through the JAK/STAT pathway, recent advances and future challenges. Gene.

[9] Hajar T., Gontijo J.R.V., Hanifin J.M. (2018). New and developing therapies for atopic dermatitis. An. Bras. Dermatol..

[10] Man G., Hu L.-Z., Elias P.M., Man M.-Q. (2018). Therapeutic benefits of natural ingredients for atopic dermatitis. Chin. J. Integr. Med..

[11] Ming J.X., Wang Z.C., Huang Y., Ohishi H., Wu R.J., Shao Y., Wang H., Qin M.Y., Wu Z.L., Li Y.Y. (2020). Fucoxanthin extracted from *Laminaria Japonica* inhibits metastasis and enhances the sensitivity of lung cancer to Gefitinib. J. Ethnopharmacol..

[12] Chengkui Z., Tseng C.K., Junfu Z., Chang C.F. (1984). Chinese seaweeds in herbal medicine. Hydrobiologia.

[13] Lee J.-Y., Lee M.-S., Choi H.-J., Choi J.-W., Shin T., Woo H.-C., Kim J.-I., Kim H.-R. (2013). Hexane fraction from *Laminaria japonica* exerts anti-inflammatory effects on lipopolysaccharide-stimulated RAW 264.7 macrophages via inhibiting NF-kappaB pathway. Eur. J. Nutr..

[14] Oh J.-H., Kim J., Lee Y. (2016). Anti-inflammatory and anti-diabetic effects of brown seaweeds in high-fat diet-induced obese mice. Nutr. Res. Pract..

[15] Fang Q., Wang J.-F., Zha X.-Q., Cui S.-H., Cao L., Luo J.-P. (2015). Immunomodulatory activity on macrophage of a purified polysaccharide extracted from *Laminaria japonica*. Carbohydr. Polym..

[16] Lee K.-S., Cho E., Weon J.B., Park D., Fréchet M., Chajra H., Jung E. (2020). Inhibition of UVB-Induced inflammation by *Laminaria japonica* extract via regulation of nc886-PKR pathway. Nutrients.

[17] Hwang Y.H., Ma J.Y. (2018). Preventive effects of an UPLC-DAD-MS/MS fingerprinted hydroalcoholic extract of *Citrus aurantium* in a mouse model of ulcerative colitis. Planta Med..

[18] Pantami H.A., Ahamad Bustamam M.S., Lee S.Y., Ismail I.S., Mohd Faudzi S.M., Nakakuni M., Shaari K. (2020). Comprehensive GCMS and LC-MS/MS metabolite profiling of *Chlorella vulgaris*. Mar. Drugs.

[19] Kang M.C., Cho K., Lee J.H., Subedi L., Yumnam S., Kim S.Y. (2019). Effect of resveratrol-enriched rice on skin inflammation and pruritus in the NC/Nga mouse model of atopic dermatitis. Int. J. Mol. Sci..

[20] Leung D.Y.M., Boguniewicz M., Howell M.D., Nomura I., Hamid Q.A. (2004). New insights into atopic dermatitis. J. Clin. Investig..

[21] Hou D.D., Di Z.H., Qi R.Q., Wang H.X., Zheng S., Hong Y.X., Guo H., Chen H.D., Gao X.H. (2017). Sea Buckthorn (*Hippophaë rhamnoides* L.) oil improves atopic dermatitis-like skin lesions via inhibition of NF-κB and STAT1 activation. Skin Pharmacol. Physiol..

[22] Jeong S.-I., Choi B.-M., Jang S.I. (2010). Sulforaphane suppresses TARC/CCL17 and MDC/CCL22 expression through heme oxygenase-1 and NF-κB in human keratinocytes. Arch. Pharm. Res..

[23] Yang J.-H., Hwang Y.-H., Gu M.-J., Cho W.-K., Ma J.Y. (2015). Ethanol extracts of *Sanguisorba officinalis* L. suppress TNF-α/IFN-γ-induced pro-inflammatory chemokine production in HaCaT cells. Phytomedicine.

[24] Lim H.-S., Seo C.-S., Jin S.-E., Yoo S.-R., Lee M.-Y., Shin H.-K., Jeong S.-J. (2016). Ma Huang tang suppresses the production and expression of inflammatory chemokines via downregulating STAT1 phosphorylation in HaCaT keratinocytes. Evid. Based Complement. Alternat. Med..

[25] Barker J.N., Jones M.L., Mitra R.S., Crockett-Torabe E., Fantone J.C., Kunkel S.L., Warren J.S., Dixit V.M., Nickoloff B.J. (1991). Modulation of keratinocyte-derived interleukin-8 which is chemotactic for neutrophils and T lymphocytes. Am. J. Pathol..

[26] Yang J.-H., Yoo J.-M., Lee E., Lee B., Cho W.-K., Park K.-I., Ma J.Y. (2018). Anti-inflammatory effects of *Perillae herba* ethanolic extract against TNF-α/IFN-γ-stimulated human keratinocyte HaCaT cells. J. Ethnopharmacol..

[27] Kwon D.-J., Bae Y.-S., Ju S.M., Goh A.R., Youn G.S., Choi S.Y., Park J. (2012). Casuarinin suppresses TARC/CCL17 and MDC/CCL22 production via blockade of NF-κB and STAT1 activation in HaCaT cells. Biochem. Biophys. Res. Commun..

[28] Park J.-W., Lee H.-S., Lim Y., Paik J.-H., Kwon O.-K., Kim J.-H., Paryanto I., Yunianto P., Choi S., Oh S.-R. (2018). *Rhododendron album* blume extract inhibits TNF-α/IFN-γ-induced chemokine production via blockade of NF-κB and JAK/STAT activation in human epidermal keratinocytes. Int. J. Mol. Med..

[29] Pan Z., Zhou Y., Luo X., Ruan Y., Zhou L., Wang Q., Yan Y.J., Liu Q., Chen J. (2018). Against NF-κB/thymic stromal lymphopoietin signaling pathway, catechin alleviates the inflammation in allergic rhinitis. Int. Immunopharmacol..

[30] Park J.-H., Kim M.-S., Jeong G.-S., Yoon J. (2015). *Xanthii fructus* extract inhibits TNF-α/IFN-γ-induced Th2-chemokines production via blockade of NF-κB, STAT1 and p38-MAPK activation in human epidermal keratinocytes. J. Ethnopharmacol..

[31] Goh K.C., Haque S.J., Williams B.R. (1999). p38 MAP kinase is required for STAT1 serine phosphorylation and transcriptional activation induced by interferons. EMBO J..

[32] Qi X.-F., Kim D.-H., Yoon Y.-S., Li J.-H., Song S.-B., Jin D., Huang X.-Z., Teng Y.-C., Lee K.-J. (2009). The adenylyl cyclase-cAMP system suppresses TARC/CCL17 and MDC/CCL22 production through p38 MAPK and NF-κB in HaCaT keratinocytes. Mol. Immunol..

[33] Vestergaard C., Yoneyama H., Matsushima K. (2000). The NC/Nga mouse: A model for atopic dermatitis. Mol. Med. Today.

[34] Jin H., He R., Oyoshi M., Geha R.S. (2009). Animal models of atopic dermatitis. J. Investig. Dermatol..

[35] Sehra S., Tuana F.M.B., Holbreich M., Mousdicas N., Kaplan M.H., Travers J.B. (2008). Clinical correlations of recent developments in the pathogenesis of atopic dermatitis. An. Bras. Dermatol..

[36] Park J.N., Ali-Nehari A., Woo H., Chun B. (2012). Thermal stabilities of polyphenols and fatty acids in *Laminaria japonica* hydrolysates produced using subcritical water. Korean J. Chem. Eng..

[37] Ziboh V.A., Miller C.C., Cho Y. (2000). Metabolism of polyunsaturated fatty acids by skin epidermal enzymes: Generation of antiinflammatory and antiproliferative metabolites. Am. J. Clin. Nutr..

[38] Kaczmarski M., Cudowska B., Sawicka-Zukowska M., Bobrus-Chociej A. (2013). Supplementation with long chain polyunsaturated fatty acids in treatment of atopic dermatitis in children. Postepy Dermatol. Alergol..

[39] Tang L., Li X., Wan L., Wang H., Mai Q., Deng Z., Ding H. (2020). Ameliorative effect of orally administered different linoleic acid/α-linolenic acid ratios in a mouse model of DNFB-induced atopic dermatitis. J. Funct. Foods.

[40] Yoshida S., Yasutomo K., Watanabe T. (2016). Treatment with DHA/EPA ameliorates atopic dermatitis-like skin disease by blocking LTB4 production. J. Med. Investig..

